# Chronic pancreatitis complicated by pancreatico-pleural fistula leading to black pleural effusion: a case report

**DOI:** 10.1186/s12890-026-04123-3

**Published:** 2026-04-01

**Authors:** Wenwen Yu, Jincong Wang, Xie Zhang, Yunlei Li, Legui Zheng

**Affiliations:** Department of Respiratory and Critical Care Medicine, Affiliated Yueqing Hospital of Wenzhou Medical University, Wenzhou, 325600 Zhejiang China

**Keywords:** Pancreaticopleural fistula, Pleural effusion, Chronic pancreatitis

## Abstract

**Background:**

Pancreaticopleural fistula, a rare but serious complication of chronic pancreatitis, typically presents with recurrent massive hemorrhagic pleural effusion. Respiratory symptoms dominate the clinical picture, while abdominal signs are often subtle.

**Case presentation:**

A 43-year-old male was admitted with a 5-day history of chest tightness. Chest computed tomography (CT) revealed massive left pleural effusion. Thoracentesis yielded black pleural fluid, but routine analysis was inconclusive. Given the patient’s history of alcohol abuse and chronic abdominal distension, an abdominal CT was performed, showing atrophy and multiple calcifications in the body and tail of the pancreas. Serum and pleural fluid amylase levels were measured at 354 U/L and > 6000 U/L, respectively. Contrast-enhanced upper abdominal CT and magnetic resonance cholangiopancreatography‌ (MRCP) further demonstrated a fistula extending from the pancreas to the left pleural cavity. The patient was diagnosed with chronic pancreatitis, pancreaticopleural fistula, and pancreas-related pleural effusion. Treatment included thoracic drainage, parenteral nutrition, and intravenous administration of somatostatin and omeprazole to reduce pancreatic secretion. The patient’s condition improved significantly.

**Conclusions:**

Patients with chronic pancreatitis complicated by pancreaticopleural fistula may present predominantly with respiratory symptoms and lack significant abdominal manifestations, which can lead to missed or delayed diagnosis. A markedly elevated amylase level in pleural fluid serves as a crucial diagnostic clue. Confirmation of pancreaticopleural fistula can be achieved through upper abdominal imaging evaluation.

## Background

Pancreaticopleural fistula (PPF) is a rare but serious complication of chronic pancreatitis. It may present with recurrent, massive hemorrhagic pleural effusion, predominantly featuring respiratory symptoms such as respiratory distress or chest tightness with minimal abdominal manifestations, often leading to missed or misdiagnosis [1, 2]. This article reports a case of chronic pancreatitis complicated by PPF, which initially manifested as chest tightness and resulted in massive black pleural effusion. By reviewing relevant literature, we summarize the etiology, clinical features, diagnosis, and treatment of PPF, aiming to enhance clinicians’ awareness of uncommon causes in the differential diagnosis of pleural effusion and to emphasize the critical role of measuring pleural fluid amylase levels and imaging studies such as MRCP in diagnosing pancreaticopleural fistula.

## Case presentation

**Fig. 1:**
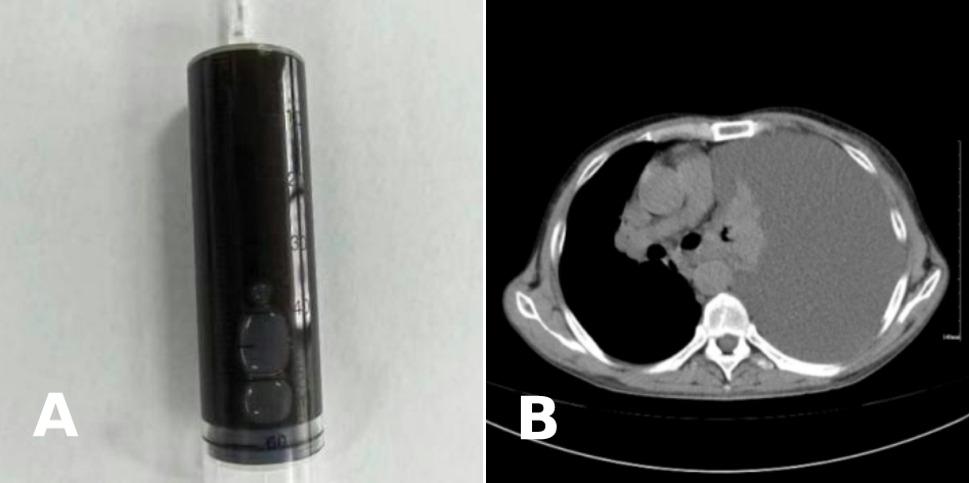
(**A**) Black pleural fluid obtained via thoracic drainage from the left pleural cavity. (**B**) Axial chest CT image demonstrating massive left-sided pleural effusion with obscuration of the left lung and mediastinal shift to the right

A 43-year-old male was admitted due to chest tightness for 5 days, accompanied by dyspnea and abdominal distension. He denied other symptoms, including abdominal pain, fever, or cough. Chest computed tomography (CT) on admission revealed massive left pleural effusion. Initial thoracentesis revealed copious black pleural fluid (Fig. 1A). Physical examination on admission revealed: an alert mental status, temperature 36.4 °C, respiratory rate 20 breaths per minute, pulse 104 beats per minute, blood pressure 135/77 mmHg, and peripheral oxygen saturation (SpO₂) 99%. Auscultation of the right lung revealed coarse breath sounds without rales, in contrast to the absent breath sounds on the left. Percussion of the left hemithorax demonstrated dullness. Heart sounds were regular without murmurs. The abdomen was soft and non-tender, without hepatosplenomegaly, rebound tenderness, or Murphy’s sign, or shifting dullness. There was no lower extremity edema. Laboratory findings showed: pleural fluid: rivalta test positive, specific gravity 1.028, red blood cell 532,000/µL, nucleated cells 585 × 10⁶/L (neutrophils 35%, lymphocytes 23%, and macrophages 42%). Biochemistry examination showed: total protein 38.3 g/L, adenosine deaminase 20.4 U/L, lactate dehydrogenase 759 U/L. Carcinoembryonic antigen was 0.58 ng/mL. Bacterial cultures, tuberculosis X-pert, and repeated cytopathology were all negative. Blood tests showed: white blood cell 4.52 × 10⁹/L, neutrophils 65.2%, hemoglobin 116 g/L, platelet 297 × 10⁹/L; C-reactive protein 11.2 g/L. Procalcitonin, liver/renal function, electrolytes, cardiac enzymes, coagulation, tumor markers, infectious serology, immunoglobulin G4, brain natriuretic peptide, and interferon gamma release assay were largely normal. Chest CT showed massive left pleural effusion (Fig. 1B).

**Fig. 2:**
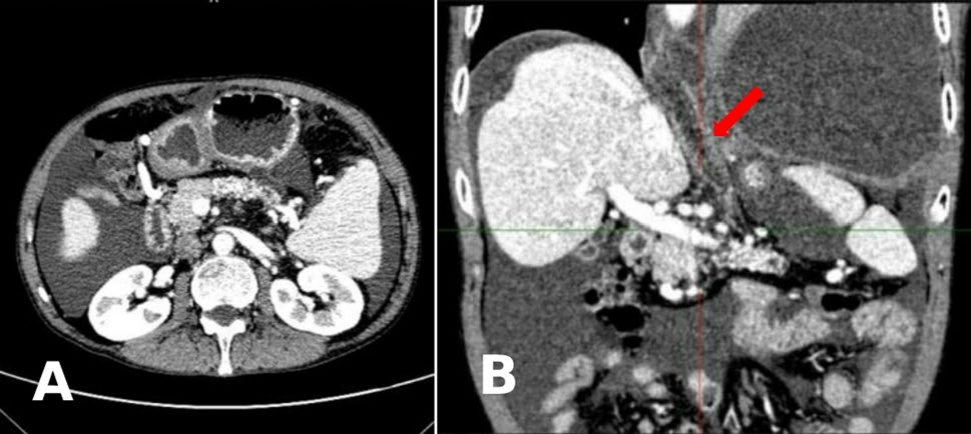
(**A**) Contrast-enhanced axial CT of the upper abdomen showing pancreatic atrophy with multiple clustered dense calcifications in the body and tail. (**B**) Coronal reconstruction image of contrast-enhanced upper abdominal CT demonstrating a tubular fluid-density tract (red arrow) extending upward from the pancreatic body through the esophageal hiatus toward the left pleural cavity

**Fig. 3:**
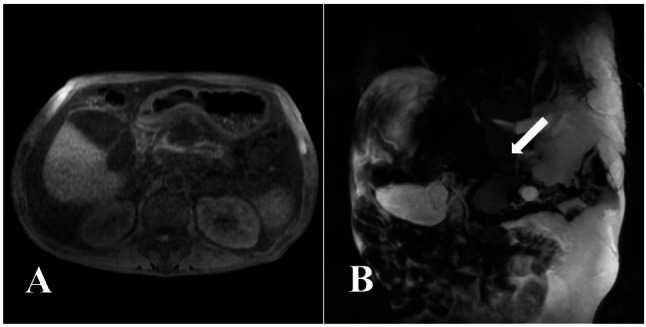
(**A**) MRI T1-weighted imaging sequence reveals an irregularly shaped cystic fluid signal in the pancreatic body, accompanied by volume loss and heterogeneous signal intensity in the body and tail. (**B**) Coronal MRCP demonstrates a tubular fluid-signal tract (white arrow) extending superiorly from the pancreatic body through the esophageal hiatus into the left pleural cavity

For an initial diagnosis of pleural effusion of unknown origin, the patient received piperacillin-tazobactam and thoracentesis. Common causes, including trauma, parapneumonic effusion, empyema, tuberculosis, and malignancy, were ruled out. Given the patient’s history of alcohol abuse (> 10 years) and persistent abdominal distension (> 2 years), on the 4th day after admission, an abdominal CT was performed, revealing atrophy and calcifications in the pancreatic body and tail. Considering the possibility of pancreas-related pleural effusion, serum and pleural fluid amylase levels were further measured on the 5th day of admission, showing results of 354 U/L (biological reference interval: 30–110 U/L) and > 6000 U/L (biological reference interval: 30–110 U/L), respectively. To further clarify the relationship between the pleural cavity and pancreatic abnormalities, the patient underwent contrast-enhanced upper abdominal CT, magnetic resonance imaging (MRI), and MRCP on the 6th day of admission. These examinations further confirmed pancreatic atrophy with calcifications (Figs. 2A and 3A) and a fistulous tract extending from the pancreatic body through the esophageal hiatus into the left pleural cavity (Figs. 2B and 3B).

The final diagnosis was chronic pancreatitis with pancreaticopleural fistula and pancreas-related pleural effusion, confirmed by gastroenterology and gastrointestinal surgery consultations. As no acute abdomen was present, conservative management was prioritized. Subsequently, the patient was kept nothing by mouth to minimize pancreatic stimulation. Total parenteral nutrition was initiated for nutritional support and maintained for 8 days. Octreotide was administered subcutaneously for 5 days to reduce pancreatic exocrine secretion. Intravenous omeprazole was also given to suppress gastric acid. Thoracic drainage was maintained. After 8 days of treatment, the pleural drainage decreased significantly from an initial volume of approximately 800 mL/day to less than 50 mL/day. The patient’s respiratory symptoms (chest tightness and dyspnea) resolved. Oral intake was restarted on hospital day 14 with a clear liquid diet, following the observed reduction in drainage output and clinical improvement. The patient tolerated this well without an increase in drainage. His condition continued to improve, and he was discharged on the 16th day of admission. Within one year post-discharge, he was readmitted twice for alcohol-induced pancreatitis, which was managed conservatively. During these readmissions, CT did not show re-accumulation of pleural effusion or obvious fistula tract. However, MRCP was not performed. He subsequently abstained from alcohol.

## Discussion

Pancreatic fistula was first defined by Cameron et al. in 1976 as an internal fistula wherein pancreatic exocrine secretions drain into body cavities instead of the duodenum [3]. Pancreaticopleural fistula (PPF) arises from inflammation or trauma leading to rupture of the main pancreatic duct or its branches into the retroperitoneal space. Pancreatic secretions then track superiorly, often through the aortic or esophageal hiatus, forming a fistula into the pleural cavity, penetrating the mediastinal pleura, and resulting in unilateral or bilateral pleural effusions [4, 5]. Fistulae occur in 3–7% of patients with chronic pancreatitis, manifesting as pancreatic pleural or ascitic effusions [6]. In contrast, pleural effusions associated with acute pancreatitis are typically reactive, self-limiting, and occur in only 3–17% of cases [7]. In adults, PPF most commonly complicates chronic alcoholic pancreatitis; whereas in children, it is frequently associated with idiopathic pancreatitis or structural anomalies [8]. Given its rarity, current evidence is largely limited to case reports.

Pancreatic pleural effusions are often recurrent, massive, and hemorrhagic. However, several reports, including the present case, describe black pleural effusion [9, 10]. Previous literature reports suggest that the black pleural effusion most likely due to haemolysis and the presence of haemosiderinladen macrophages after a massive bleed into the pleura [11–13]. In this patient, the black appearance of the effusion and the markedly elevated pleural fluid RBC count (532,000/µL) both indicate that the effusion accumulated over an extended period following a hemorrhagic event well before clinical presentation. A markedly elevated pleural fluid amylase level is a simple and reliable diagnostic indicator, typically exceeding 1000 U/L. A level greater than 5000 U/L is considered highly specific and may be diagnostic for PPF [14]. Notably, elevated pleural amylase can also occur in malignancy or esophageal rupture; these were ruled out in this case through clinical history, CT imaging, and cytopathological analysis. While the sensitivity of CT for detecting pancreato-mediastinal fistulas is approximately 50%, MRCP offers a higher sensitivity of up to 80%, making it the preferred non-invasive modality for identifying PPF [15, 16]. Patients with suspected PPF should undergo thorough imaging, including abdominal ultrasound, CT, MRCP, and/or endoscopic retrograde cholangiopancreatography (ERCP). In our case, both contrast-enhanced upper abdominal CT and MRCP revealed a thick-walled cystic lesion above the pancreas communicating with the left pleural space, which confirmed the diagnosis of PPF. In this case, the left pleural effusion likely resulted from pseudo pancreatic cyst secondary to chronic pancreatitis, with pancreatic fluid tracking into the pleural space through a pancreaticopleural fistula.

There remains no clear consensus on the optimal method or timeline for managing PPF. Initial management is conservative, involving closed thoracic drainage, the use of broadspectrum antibiotics, proton pump inhibitors, parenteral nutrition. The use of proton pump inhibitors and parenteral nutrition was aimed at reducing gastric acid secretion, thereby decreasing pancreatic fluid output [6]. In addition, the administration of somatostatin analogs has been reported to reduce the output of pancreatic juice [17, 18]. For refractory cases, ERCP with therapeutic interventions, such as sphincterotomy, stone extraction, pancreatic stenting, nasopancreatic drainage, or stricture dilation, is recommended [18]. Surgery, including cyst resection or pancreaticojejunostomy, is reserved for failures of endoscopic therapy [19]. Our patient presented initially with chest tightness and massive black bloody pleural effusion, leading to admission under respiratory medicine. Despite a significant alcohol history, he had no typical abdominal symptoms, underscoring the insidious nature of chronic pancreatitis. Unexplained black hemorrhagic effusion prompted measurement of pleural amylase, which was significantly elevated. Subsequent abdominal CT revealed pancreatic atrophy with calcifications, and MRCP demonstrated a fistulous tract extending into the chest, consistent with PPF. The patient responded favorably to conservative medical management. When conservative measures fail, endoscopic or surgical interventions are necessary for managing pancreatic fistulas.

## Conclusions

For patients who present primarily with respiratory symptoms and lack abdominal symptoms, clinicians may overlook abdominal examinations. When confronted with unexplained hemorrhagic pleural effusion, clinicians should remain alert to the possibility of PPF. This is particularly important for patients with risk factors for chronic pancreatitis. Pleural fluid amylase measurement is a useful initial test. Abdominal contrast-enhanced CT or MRCP can subsequently confirm the presence of a fistula. First-line treatment is conservative medical management. Refractory cases may require endoscopic or surgical intervention.

## Data Availability

Data is provided within the manuscript.

## Abbreviations

PPF: Pancreaticopleural fistula
CT: Computed tomography
MRCP: Magnetic resonance cholangiopancreatography
MRI: Magnetic resonance imaging
ERCP: Endoscopic retrograde cholangiopancreatography
